# The Dynamics of Connexin Expression, Degradation and Localisation Are Regulated by Gonadotropins during the Early Stages of In Vitro Maturation of Swine Oocytes

**DOI:** 10.1371/journal.pone.0068456

**Published:** 2013-07-04

**Authors:** Nicolas Santiquet, Claude Robert, François J. Richard

**Affiliations:** Centre de recherche en biologie de la reproduction, Département des sciences animales, FSAA, Université Laval, Québec, Québec, Canada; University of Nevada School of Medicine, United States of America

## Abstract

Gap junctional communication (GJC) plays a primordial role in oocyte maturation and meiotic resumption in mammals by directing the transfer of numerous molecules between cumulus cells and the oocyte. Gap junctions are made of connexins (Cx), proteins that regulate GJC in numerous ways. Understanding the dynamic regulation of connexin arrangements during in vitro maturation (IVM) could provide a powerful tool for controlling meiotic resumption and consequently *in vitro* development of fully competent oocytes. However, physiological events happening during the early hours of IVM may still be elucidated. The present study reports the dynamic regulation of connexin expression, degradation and localization during this stage. Cx43, Cx45 and Cx60 were identified as the main connexins expressed in swine COC. Cx43 and Cx45 transcripts were judged too static to be a regulator of GJC, while Cx43 protein expression was highly responsive to gonadotropins, suggesting that it might be the principal regulator of GJC. In addition, the degradation of Cx43 expressed after 4.5 h of IVM in response to equine chorionic gonadotropin appeared to involve the proteasomal complex. Cx43 localisation appeared to be associated with GJC. Taken together, these results show for the first time that gonadotropins regulate Cx43 protein expression, degradation and localisation in porcine COC during the first several hours of IVM. Regulation of Cx43 may in turn, via GJC, participate in the development of fully competent oocytes.

## Introduction

Gap junctional communication (GJC) plays key roles in numerous tissues and cell types, including ovaries, follicles and cumulus oocyte complex (COC). Indeed, GJC is involved in several physiological and pathological processes such as the cell cycle, cell proliferation and differentiation, cell survival and death, tissue homeostasis, arrhythmia, tumour development, cancer and neurodegenerative diseases (reviewed in [1], [2], [3], [4], [5]). GJC also plays a primordial role in oocyte maturation and meiotic resumption in mammals [6], [7].

Gap junctions are channels that allow direct exchange of ions and small molecules (≤1 kDa) between adjacent cells. Their formation results from mutual docking of plasma membrane hemi-channels called connexons. Each connexon is composed of six trans-membrane protein molecules called connexins. Connexins are expressed ubiquitously in animal cells, except in differentiated skeletal muscle cells, erythrocytes, and mature sperm cells [8]. About 20 connexins have been identified in mammals. Eight of these, namely Cx26, Cx30.3, Cx32, Cx37, Cx40, Cx43, Cx45 and Cx57 (or ortholog Cx60 in swine) are expressed in the mammalian ovary [6], [9], [10]. Connexons can be composed of one or more types of connexin, which interact with each other to form homomeric, heteromeric, or heterotypic channels [11]. The ability of the cell to mix gap junction connexin content in this manner increases possibilities for the regulation of specific permeability [12], [13].

Connexin 26 has been detected in oocytes, granulosa cells, and theca cells of several species [6], [10]. Cx26 knockout mice embryos die 11 days post coïtum, although no specific function of this connexin has been determined in the ovary [14], [15]. Using an in situ hybridization technique, connexin 30.3 has been found in porcine follicle, theca cells, granulosa cells and cumulus cells [16]. Connexin 32 is also expressed in the porcine ovary, specifically in theca cells [16]. Cx32 knockout mice are viable and fertile [17]. Human chorionic gonadotropin (hCG) has been shown to induce down regulation of Cx32 in mouse cumulus–oocyte complexes, suggesting that Cx32 probably plays a role in the response to gonadotropins [18]. In cattle, Cx32 is expressed in granulosa cells of atretic but not healthy follicles [19], suggesting that Cx32 could be involved in apoptosis. However, the exact role of Cx32 in follicular function remains to be deciphered. A connexin-37-knockout mouse shows an arrest of folliculogenesis at the early antral stage of follicle growth [20]. The specific cell type that expresses connexin 37 is not known in all species. In mice, this expression occurs in the oocyte, where it allows the formation of heteromeric/heterotypic channels between the oocyte and cumulus cells, which express connexins 43 and 45. However, connexin 37 has also been shown to be expressed in the cumulus cells in the corona radiata, allowing the formation of homomeric/homotypic channels between cumulus cells and the oocyte [6], [21]. Connexin 37 has not yet been detected in the porcine ovary. Connexin 40 is known to be expressed in the ovary, but only in blood vessels in the ovarian stroma [22]. Connexin 43 is the predominant connexin expressed in granulosa and cumulus cells within the follicles of numerous species, including swine [6], [23]. Reports of its expression in oocytes are still debated [18], [24]. It is the most abundant connexin in the mouse follicle. Using Cx43 knockout mice model, it has been shown that folliculogenesis does not proceed beyond the primary follicular stage, and oocyte growth is delayed [25], [26]. Connexin 45 expression has been observed in pig, mouse and rat oocytes, granulosa cells and cumulus cells [9], [22], [27], [28]. Expression of connexin 60, the ortholog of mouse Cx57, has been shown in porcine cumulus cells and oocytes, while Cx57 is expressed at low levels in mouse ovaries [28], [29], [30].

*In vivo*, oocytes are held in meiotic prophase arrest by a chemical signal that flows in from surrounding somatic cells through gap junctions. According to the most recent signal transduction model in the oocyte, it consists essentially of cyclic adenosine monophosphate and cyclic guanosine monophosphate [31], [32], [33]. Following the luteinizing hormone surge, the GJCs turn off the flow of cyclic nucleotides, thus triggering meiotic resumption [31], [34], [35]. Removal of the cumulus-oocyte complex from the follicle also causes meiosis to resume spontaneously [36]. *In vitro*, meiotic resumption follows the loss of GJC capacity in COCs [37], [38]. Oocyte maturation and meiotic resumption can be promoted with the use of a GJC inhibitor [39], [40]. GJC has also been found to play a major role in the regulation of chromatin remodelling and transcription during the early stages of in vitro maturation (IVM) of bovine oocytes [41]. In swine, spontaneous oocyte meiosis resumption or germinal vesicle breakdown (GVBD) is substantially slower than in any other mammals. Indeed, swine oocyte remains in complete meiotic arrest during the first 18 hours of IVM, in contrast to rat, bovine, and human, which take 1, 6, and 12 h, respectively, to resume meiosis [42], [43], [44]. Due to this extent period, swine is a powerful animal model to decipher the signalling cascade of meiotic resumption.

We have demonstrated recently that in swine, gonadotropins can regulate the effectiveness of GJC between cumulus cells during the first several hours of IVM [45]. We therefore hypothesized that modulation of GJC can be explained in terms of changes in the total number of junctions, junction localization, opening frequency or gap junction pore size [13], [46], [47], [48]. The present study was undertaken to determine whether changes in connexin expression, degradation, and localization during the first 8.5 h of IVM are correlated with the previously observed dynamics of GJC [45].

We show that Cx43 and Cx45 are the main connexins expressed in porcine cumulus cells. Regulation of the transcription of these two connexins does not appear to affect GJC, although de novo transcription is necessary. Connexin 43 protein expression is highly regulated by gonadotropins and appears to follow GJC dynamics. Furthermore, Cx43 down-regulation in response to equine chorionic gonadotropin appears to occur through the proteasomal complex after 4.5 h of IVM. Finally, we show that proper localisation of junctions likely contributes to GJC regulation.

## Materials and Methods

### Chemicals

Unless otherwise stated, all chemicals were purchased from Sigma Chemical Co. (St. Louis, MO).

### Ovaries and COC Collections

Pre-pubertal pig ovaries were collected at the local slaughterhouse, placed in saline (0.9% NaCl) containing 100,000 IU/L penicillin G, 100 mg/L streptomycin and 250 mg/L amphotericin B (Invitrogen, product no. 15140) and maintained at 34°C, as described previously [49]. Upon arrival in the laboratory, they were rinsed twice with this solution. Follicles between 2 and 6 mm in diameter were punctured using a syringe with an 18-gauge needle and pooled follicle contents were placed in a 50 ml flacon tube. After sedimentation, COCs were washed twice in centrifuged porcine follicular fluid (PFF), recovered using a stereomicroscope and then washed three times in HEPES-buffered Tyrode medium containing 0.01% (w/v) polyvinyl alcohol. COCs with at least three layers containing compact and clear cumulus cells were selected. These were allowed to mature, and then 50 were washed three times in polyvinyl alcohol then once in PBS containing D-glucose (1 g/l) and sodium pyruvate (0.11 g/l) and then placed in a micro-centrifuge tube in PBS-glucose/pyruvate and centrifuged for 2 min at 5000 rpm. Supernatant was removed and the pellet was flash frozen in liquid nitrogen and placed at −80°C until processing using the PCR or Western blot protocol.

### In vitro Maturation (IVM)

Groups of 50 COCs were cultured in Nunclon Δ four-well dishes in 500 µl of standard porcine IVM culture medium: BSA-free NCSU 23 medium [50] containing 25 µM of 2-mercaptoethanol, 0.1 mg/ml cysteine and 10% (vol/vol) of filtered porcine follicular fluid as already described [45]. Depending on the experiment, the medium also contained 5 IU/well of human chorionic gonadotropin (hCG) and/or equine chorionic gonadotropin (PMSG/eCG, Intervet, Whitby, Ontario, Canada).

### PCR

Total RNA from COC or denuded oocyte was extracted and purified using a PicoPure RNA Isolation Kit (Life Science). After digestion with DNase (Qiagen), the concentration of extracted RNA was determined using the Nanodrop (Thermo Scientific) technique. RNA (50 ng) from independent samples was reverse transcribed using a qScript Flex cDNA Synthesis Kit (Quanta Biosciences) with oligo-dT primer. PCR was performed using a FastStart Taq DNA Polymerase kit (Roche Diagnostics). Specific primers for connexin were designed using PrimerQuest (Integrated DNA Technologies) or by multiple sequence alignment of different species sequences (mouse, human, cattle, rat) using ClustalW2® software (http://www.ebi.ac.uk/Tools/msa/clustalw2/). Primer sequences, product size, annealing temperature, and accession numbers are shown in Table 1. The following cycling conditions were used for all amplifications: 15 minutes at 95°C, followed by 35 cycles of 1 minute at 95°C, 1 minute at 55°C and 1 minute at 72°C, followed by 10 minutes at 72°C. Amplicons were visualized by electrophoresis in 1% agarose gel and staining with ethidium bromide. Bands were then removed for sequencing with a StrataPrep DNA Gel Extraction kit (Agilent). Sequencing was performed at the Université Laval sequencing laboratory. Amplicon sequences were BLAST analyzed for identification. Those with greater than 95% similarity to the predicted sequence were presumed primer-specific.

**Table 1:** Sequence, product size, annealing temperature, and accession number of primers used for PCR and RT-qPCR analysis.

Gene symbol	Name	Accession number	Forward sequence 5′-3′	Reverse sequence 5′-3′	Productsize (bp)	Annealingtemperature(°C)
**TBP1**	TATA Binding Box Protein 1	XM_003361418	AACAGTTCAGTAGTTATGAGCCAGA	AGATGTTCTCAAACGCTTCG	153	55°C
**HPRT1**	Hypoxanthine phosphoribosyltransferase 1	NM_001032376	GGACTTGAATCATGTTTGTG	CAGATGTTTCCAAACTCAAC	91	55°C
**RPL4**	Ribosomal protein L4	XM_003121741	CAAGAGTAACTACAACCTTC	GAACTCTACGATGAATCTTC	122	55°C
**Cx43**	Connexin 43	NM_001244212	ACTGAGCCCCTCCAAAGACT	GCTCGGCACTGTAATTAGCC	191	55°C
**Cx45**	Connexin 45	NM_001097519	TGTATGGCTTCCAAGTCCACCCAT	TAAGCAGTAGGCAAAGGCCTGTCA	144	55°C
**Cx60**	Connexin 60	NM_010289NM_001173508NM_032602XM_002690190	GGCATTGAGGATGAAAGGGG	GAAGCCATTGTTGTACCTAGCC	378	55°C

For RT-quantitative PCR (RT-qPCR) analysis, LightCycler 480 SYBR Green I Master and a LightCycler 480 device (Roche) were used. A five-point standard curve of amplicon diluted from 1 pg to 0.1 fg was used for real-time quantification of the PCR output of each primer pair. The GeNORM factor [51] from expression values of three reference genes (TBP1, HPRT1, RPL4) [52] was used for data normalization. Reference genes primers spanning at least one intron were designed to minimize inaccuracies due to possible genomic contamination [52]. Primer sequences, product size, annealing temperature, and accession numbers of the sequences used to design the primers are provided in Table 1.

### Western Blot

Sample buffer (62.5 mM Tris-HCl pH 6.8, 10% glycerol [vol/vol], 2% sodium dodecyl sulfate [wt/vol], 0.005% bromophenol blue [wt/vol]) was added to a micro-centrifuge tube containing 50 COCs to extract proteins. Briefly, COCs were lysed by multiple pipetting, then heat at 95°C for 3 min and centrifuge at 13000 RPM (4°C) for 20 min. Next, supernatant was removed and placed in a new tube, heated at 95°C for 3 min and centrifuge at 13000 RPM (4°C) for 4 min. Supernatant volume corresponding to 30 COCs supplemented with 5% β-mercaptoethanol was loaded onto 10% polyacrylamide gel for electrophoresis. Separated proteins were then transferred onto PVDF membranes (IPVH00010; Millipore, Temecula, CA). Membranes were blocked for 1 hour with Tris-buffered saline plus Tween 20 (TBST: 150 mM NaCl, 20 mM Tris-HCl pH 7.4, 0.1% [vol/vol] Tween 20) containing 5% (wt/vol) non-fat dry milk. The first hybridization was performed overnight at 4°C with the primary antibody, mouse anti-Cx43 diluted 1∶2000 in TBST (MAB3067; Millipore, Temecula, CA). Membranes were then hybridized for 45 min with horseradish peroxidase-conjugated goat anti-mouse Ig diluted 1∶10,000 in TBST (12-349; Millipore, Temecula, CA). Proteins were detected using an ECL kit (GE Healthcare) and Fusion FX7 reader from MBI Lab Equipment using Fusion software. Membranes were then stripped for 1 hour to remove all antibodies in order to allow subsequent hybridization with anti-tubulin (stripping buffer: glycine-HCl 25 mM, SDS 1%, pH 2.0). Membranes were blocked for 1 hour with TBST containing 5% (wt/vol) non-fat dry milk, hybridized with mouse anti-tubulin diluted 1∶50,000 (T4216; Sigma) for 1 hour and then exposed for 1 hour with the horseradish peroxidase-conjugated anti-Ig described above. Proteins were detected as previously described above. The relative expression of Cx43 was quantified with normalization against tubulin. Densitometric quantification of signals was achieved using BioD analysis software.

### Immunofluorescence

Three hundred COCs were fixed in 4% paraformaldehyde for 1 hour at room temperature, then placed at 4°C in 1% paraformaldehyde overnight and embedded in paraffin. Sections 5 µm thick were mounted on slides, de-waxed in toluene, re-hydrated and then boiled for 20 min in Tris/EDTA pH 9.0. Sections were blocked for 20 min using 10% goat serum solution, followed by incubation overnight at 4°C with mouse anti-Cx43 diluted 1∶50 (MAB3067; Millipore, Temecula, CA). Sections were blocked again the following day in 10% goat serum solution for 20 min and incubated with goat anti-mouse Ig conjugated with Alexa Fluor® 488 diluted 1∶200 (Invitrogen Alexa Fluor®; A-11029). Sections were then mounted with ProLong® Gold anti-fade reagent with DAPI (Invitrogen; P36935) and slides were viewed using a Zeiss LSM700 microscope.

### Gap-FRAP Assay

The Gap-FRAP assay was performed as described previously [45]. Briefly, this assay consisted of measuring the transfer of the fluorescent dye calcein from cells to a cell that had been laser-bleached to eliminate calcein fluorescence. COCs were washed three times in 0.01% (w/v) polyvinyl alcohol and then placed in 500 µL of PBS containing 1 µM calcein-AM and 0.05% Pluronic-127 (Invitrogen, P-3000MP) at 38°C. The dye was allowed to diffuse for 30 min and be cleaved into fluorescent calcein, after which the COCs were washed twice in 0.01% (w/v) PVA and once in PBS to remove any calcein-AM. COCs were then mounted on glass slides in PBS. A Nikon Eclipse TE2000-E inverted confocal microscope equipped with an argon laser (488 nm) was used at 60X magnification to perform photo-bleaching on specific cells and measure fluorescence recovery. Fluorescence intensity was recorded in a confocal section for the following periods: 5 pre-bleach, 3 bleaches with 100% power laser pulses at the targeted cumulus cell, and every 10 seconds during the 5 minute post-bleach period. The experiment was repeated three times, each time with three COCs and three FRAP assays on three different cumulus cells for each COC, thus producing 27 FRAP set of data for each time point.

### Analysis of FRAP Data

Fluorescence recovery in the bleached cells was expressed using the perturbation–relaxation equation described previously [53]:
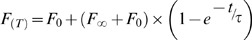
where t is the time (s) after photo-bleaching; F_(t)_ is the normalized fluorescence intensity measured at t; F**_∞_** is the asymptotic limit to which the fluorescence intensity tends; F_0_ is the estimated initial fluorescence intensity after photo-bleaching and τ is a time constant in seconds.

The dye transfer rate constant k (s^-1^) is calculated as K  = 1/τ, where K represents the transfer rate of calcein, which indicates the speed of cell–cell calcein exchange through gap junctions.

### Proteasomal Inhibition (MG132)

The proteasomal inhibitor MG132 was used at 100 µM, a dose that has been shown to inhibit meiotic resumption in porcine [1], [2]. The inhibitor was directly added to the maturation medium at 0 h.

### Statistical Analysis

Statistical analyses were done using Prism 5.00 GraphPad for Windows (GraphPad software Inc., San Diego, CA, www.graphpad.com). For real time qPCR and FRAP analysis, statistical significance was assessed by ANOVA followed by Bonferoni’s multiple comparison post hoc tests to identify individual differences between means. Differing letters indicate statistically significant differences. For western blot analysis, statistical significance was assessed by ANOVA followed by Dunnett post hoc test to identify individual differences to 0 h control column. All values are presented as the mean ± SEM. P values inferior at 0.05 were considered statistically significant.

## Results

**Figure 1:**
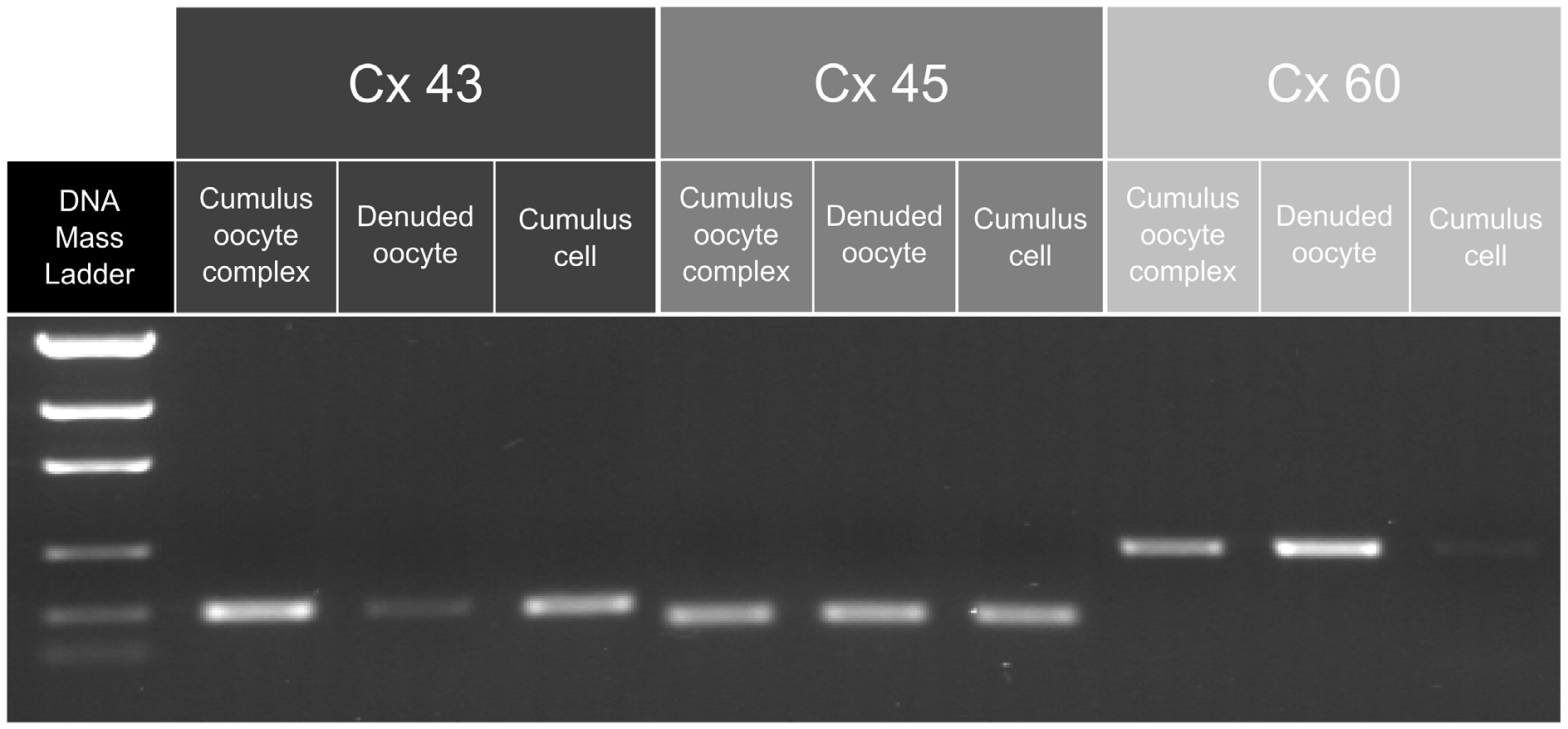
Expression of Cx43, Cx45 and Cx60 in swine COC, oocytes or cumulus cells (see Table 1 for primer and PCR product size information).

We began by identifying the main connexin expressed in porcine COC during IVM. Using a conventional PCR approach to determine first which connexin transcripts were present in the oocyte and/or cumulus cells after 4.5 h of conventional IVM (Fig. 1), we found that Cx43, Cx45 and Cx60 were strongly expressed, while Cx26 and Cx32 (data not shown) were weakly expressed. In addition, we did not detect the presence of Cx30.3 and Cx37 in spite of several attempts with different primer designs (data not shown). Cx43 is expressed abundantly in cumulus cells, Cx60 in the oocyte, and Cx45 in both cumulus cells and the oocyte.

**Figure 2:**
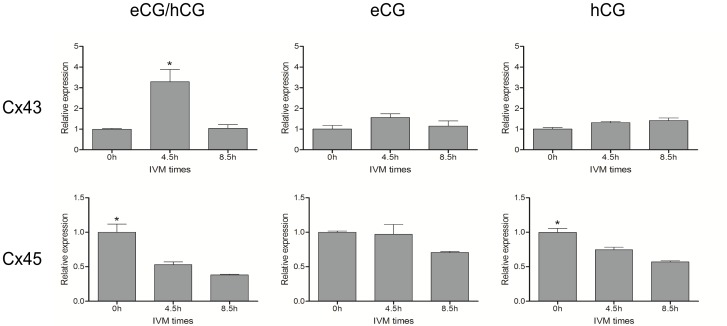
Quantification of Cx43 and Cx45 by reverse-transcription qPCR of the mRNA profile in swine COCs during the first 8.5 h of maturation. COCs were matured in conventional IVM medium plus eCG and/or hCG. Analyses were done in triplicate (pools of 50 COC each) and the amount of mRNA represents the mean ± SEM of each transcript corrected with three housekeeping genes. Different superscripts indicate significant differences (P≤0.05) based on ANOVA followed by Bonferroni’s post hoc test.

The subsequent experiment was undertaken to determine the effect of eCG and hCG on the abundance of Cx43 and Cx45 transcripts in COCs during the first 8.5 hours of IVM using the RT-qPCR approach (Fig. 2). A nearly four-fold increase in Cx43 transcripts occurred between 0 h and 4.5 h, and quantities then dropped back to the base level at 8.5 h. In contrast, Cx45 transcripts decreased to half their basal level during this time period. The experiment was then performed using either eCG alone or hCG alone. Under these conditions, Cx43 transcripts remained stable, while Cx45 transcripts decreased significantly in the presence of hCG and tends to decrease (P = 0.0913) in the presence of eCG. Based on these results, both eCG and hCG acted together to produce a four-fold increase of Cx43 transcript levels, while Cx45 transcript levels decreased independently of gonadotropins during the first 4.5 hours of IVM.

**Figure 3:**
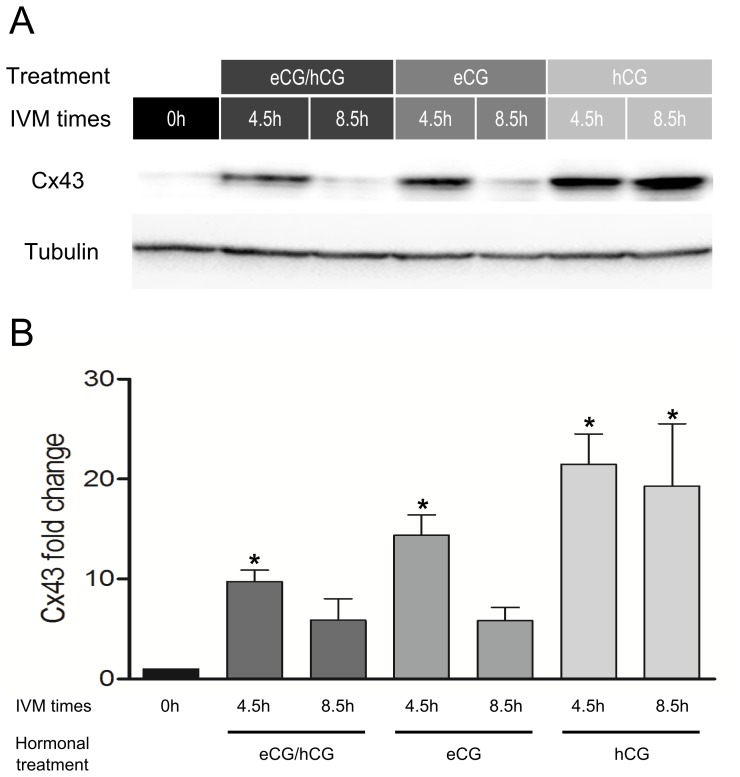
Regulation of Cx43 protein level by gonadotropins in COC during IVM. A) Immunodetection of Cx43 and α-tubulin (control) after 0, 4.5 h and 8.5 h of conventional IVM or in the presence of eCG or hCG. B) Mean densitometric ratio of Cx43/α-tubulin levels analyzed in triplicate (pool of 50 COCs) relative to 0 h. Statistical significance was assessed by ANOVA followed by Dunnett post hoc test to identify individual differences to 0 hours control column. Probabilities of P<0.05 were considered statistically significant.

We then turned our attention to the effect of gonadotropins on Cx43 protein expression (Fig. 3A). Densitometric analysis of Western blots shows that in the presence of both eCG and hCG, Cx43 increased tenfold after 4.5 h of IVM and then dropped back down to five times the basal level at 8.5 h (Fig. 3B). In the presence of eCG alone, Cx43 increased approximately 15 fold and then decreased to five times its base level at 8.5 h (Fig. 3B). In contrast, in the presence of hCG alone, Cx43 protein increased approximately 23 fold at 4.5 h and did not decrease at 8.5 h (Fig. 3B). These results suggest that gonadotropins have a large impact on Cx43 translation in porcine COC *in vitro*. They clearly show that eCG allows down-regulation of the amount of Cx43 after 4.5 h of IVM, while hCG strongly increases Cx43 protein expression, with no decrease after 4.5 h of IVM.

**Figure 4:**
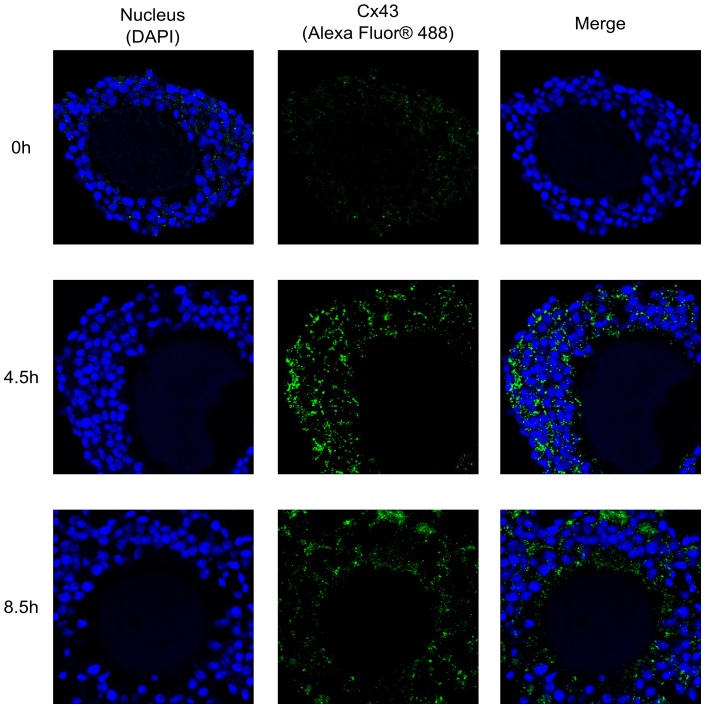
Distribution of Cx43 in COC sections. Representative immunofluorescence images of COC sections matured in classic IVM media at 0 h, 4.5 h and 8.5 h. Left to right: sections stained with DAPI to reveal the nucleus; with anti-Cx43 to reveal connexin 43 proteins (green); two stains combined.

In order to determine whether or not Cx43 segregation is a factor in the regulation of GJC between cumulus cells, we localized Cx43 in COCs sections after IVM in the presence of both eCG and hCG (Fig. 4). At 0 h, little Cx43 is found and its faint location is mostly scattered. In contrast, Cx43 is abundant at 4.5 h and is clearly forms numerous large aggregates at the cumulus cell boundary. After 8.5 h, Cx43 is less abundant, and the size and position of the aggregates have changed (Fig. 4). This result is consistent with Cx43 transcripts profiling in cumulus cells and with the Western blot results. It demonstrates that in addition to Cx43 protein expression, protein localization and formation of large aggregates are also regulated during the first hours of IVM.

**Figure 5:**
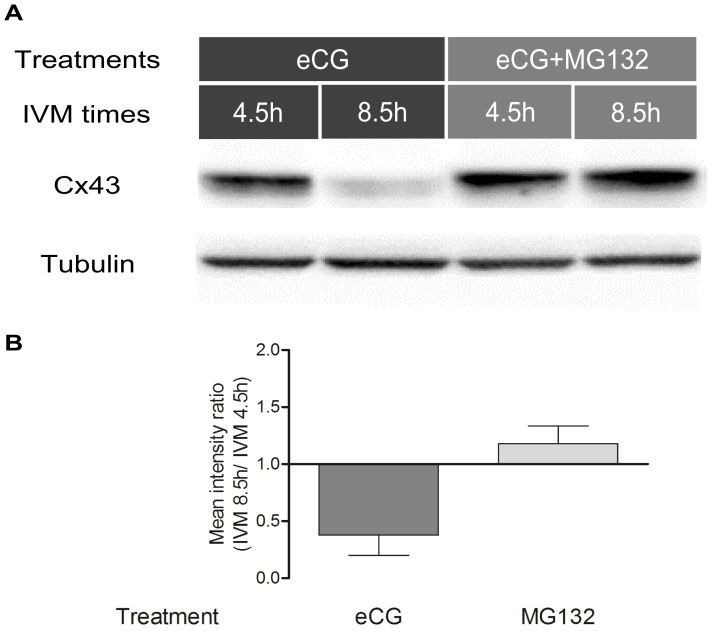
Regulation of Cx43 protein by the proteasome in COC during IVM. A) Immunodetection of Cx43 and α-tubulin (control) in COC after 4.5 h and 8.5 h of IVM in the presence of eCG or eCG and proteasomal inhibitor MG132. B) Mean densitometric ratio of Cx43 protein levels at 8.5 h and 4.5 h analyzed in triplicate (pool of 50 COCs) and corrected with α-tubulin.

To explain the drop in Cx43 protein expression observed at 8.5 h of IVM in the presence of eCG, we hypothesized that the proteasome might be involved. Based on immunodetection and densitometry, this drop did not occur in the presence of proteasome inhibitor MG132 (Fig. 5), suggesting the involvement of the proteasome in decreasing the levels of Cx43 protein reached after 4.5 h in the presence of this gonadotropin.

**Figure 6:**
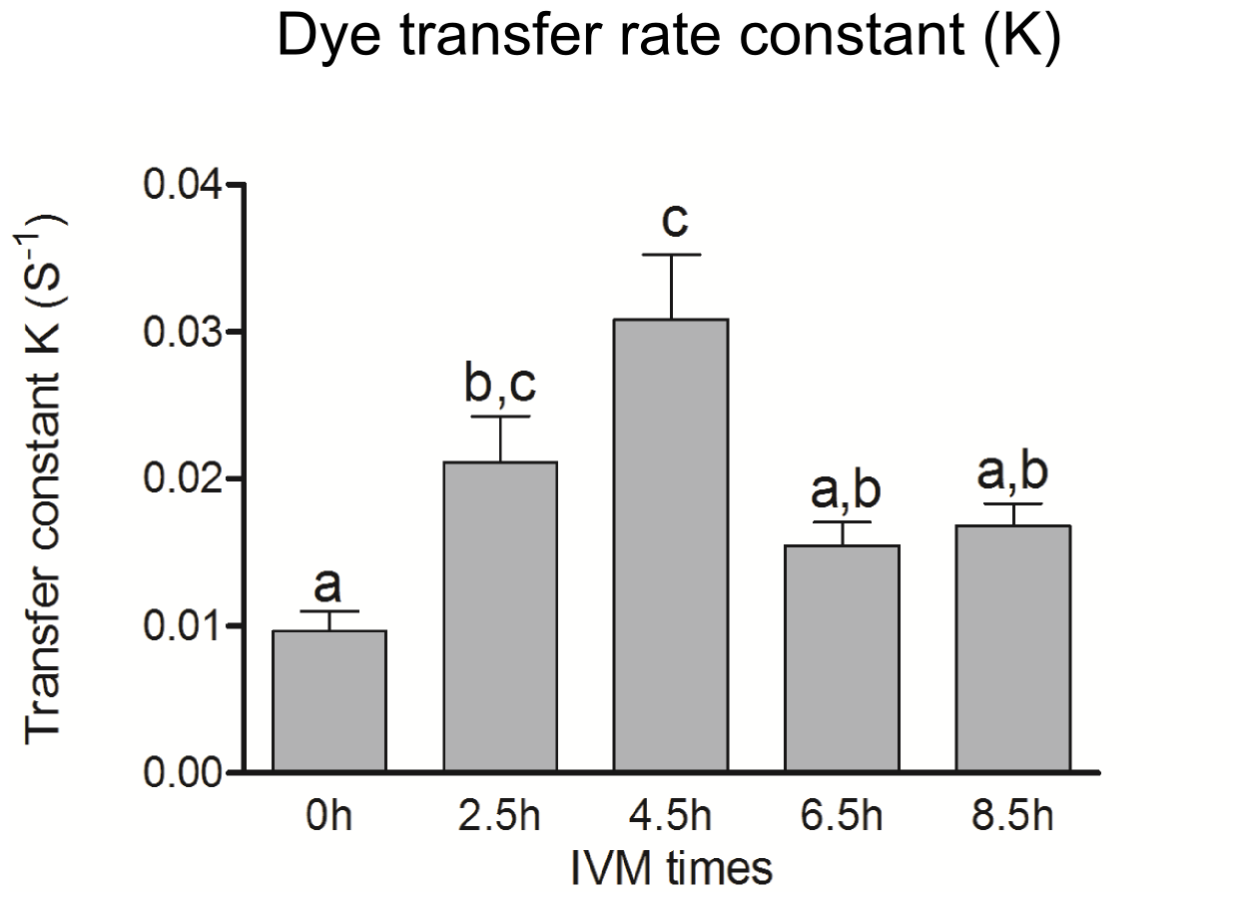
Effect of proteasomal inhibitor MG132 on GJC in cumulus cells during the first 8.5 h of IVM based on gap-FRAP assay. Data represent mean ± SEM of 27 analyses. Different superscripts indicate significant differences (P≤0.05) based on ANOVA followed by Bonferroni’s post testing.

In order to determine whether or not the proteasome could be involved in the regulation of GJC, we performed Gap-FRAP assays on cumulus cells during the first several hours of IVM in a medium containing both eCG and MG132. The dye transfer rate increased until 4.5 h and then decreased significantly at 6.5 h (Fig. 6). The overall profile of the transfer constant in the presence of eCG plus MG132 appears similar to that observed previously in the presence of eCG alone [45] and suggests that the proteasome inhibitor may prevent the drop in Cx43 protein but is unable to sustain GJC at the maximum level measured at 4.5 h.

**Figure 7:**
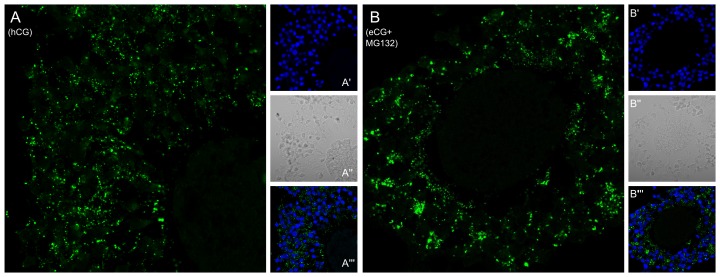
Distribution of Cx43 in COC sections. A) COC matured in hCG medium for 8.5 h. B) COC matured in eCG/MG132 medium for 8.5 h. A/B) sections stained with anti-Cx43 to reveal connexin 43 proteins (green); A’/B’) sections stained with DAPI to reveal the nucleus; A”/B”) bright-field picture of COC sections; A’’’/B’’’) DAPI and anti-Cx43 stains combined.

The purpose of the final experiment was to compare the localization of Cx43 to determine whether the pattern of immunolocalization is an indicator of the profile GJCs. For this purpose, we compared two treatments where the profile differs in GJCs at 8.5 h of IVM but where the amount of Cx43 protein was high in both situations. Indeed, GJC in COCs matured 8.5 h in medium containing hCG remained high [45] and have high amount of Cx43 protein (Fig. 3). In contrast, GJC in COCs matured 8.5 h in medium containing eCG and MG132 was reduced (Fig. 6), even though the complexes contained high amount of Cx43 protein (Fig. 5). These two conditions have high level of Cx43 at 8.5 h of IVM as measured by western blotting (Fig. 3 and 5) whereas they have different GJC profile. We thus compared the distribution patterns of Cx43 between these two conditions. The immunolocalization images in Figure 7 show that Cx43 protein are present in numerous large aggregate following treatment with eCG in the presence of MG132 (Fig. 7B). In contrast, there is fewer and smaller Cx43 aggregate following treatment with hCG (Fig. 7A). In addition, images in Figure 7 show cumulus expansion in the presence of hCG (A”) after 8.5 h of IVM, but not in the presence of eCG and MG132 (B”).

## Discussion

We have previously demonstrated that cumulus cells are crucial during the first 4 h of IVM in order for meiosis to resume properly. Indeed, removing cumulus cells affected nuclear maturation of swine oocytes, while removing them after 4 h of IVM in the presence of gonadotropins did not [54]. We have demonstrated recently that gonadotropins can regulate gap junctional communication between cumulus cells during the first 8.5 h of IVM. A major decrease in GJC is measured after 4.5 h of IVM in the presence of eCG or eCG plus hCG, but not in the presence of hCG [45]. Furthermore, it is known that oocyte maturation and embryo developmental ability *in vitro* are both decreased in the absence of gonadotropins [55]. The effect of luteinizing hormone on IVM is to produce an increase in cAMP and progesterone production and to accelerate meiotic progression to the metaphase I stage. This is consistent with our previous FRAP experiment showing no decrease in the rapid GJC observed between cumulus cells after 4.5 h of IVM in the presence of hCG and suggesting a role for GJC in this acceleration [45], [56], [57]. We therefore hypothesized that decreased GJC after 4.5 h of IVM is a characteristic of *in vitro* maturation. Molecules essential for nuclear maturation are presumably transferred actively between cells during the first 4.5 h of IVM. Variations in GJC could be explained in terms of the total number of gap junctions, junction localisation, gap opening frequency or gap junction pore size.

In the present study, we investigated the expression, degradation and localization of connexins during the first 8.5 h of IVM in order to determine whether or not such factors could be involved in modulating GJC. The connexins that compose the gap junction channel did indeed appear to be involved in the regulation of GJC. A gap junction consists of one or several types of connexin, which interact with each other to form homomeric, heteromeric or heterotypic channels [11]. The ability of the cumulus oocyte complex to form such structures no doubt determines the kinetics of permeation via gap junctions and hence the effectiveness of GJC [12]. We therefore first identified the connexins present in porcine cumulus oocyte complexes *in vitro*. Eight connexins are expressed in the mammalian ovary (reviewed in references [6], [9], [10]), namely Cx26, Cx30.3, Cx32, Cx37, Cx40, Cx43, Cx45 and Cx57 (or ortholog Cx60 in swine). Using PCR, we examined the expression of all except Cx40, which is not known to be expressed outside of ovarian stroma blood vessels [22]. We observed expression of Cx26, Cx32, Cx43, Cx45 and Cx60, but not Cx30.3 or Cx37. Of these latter two, the former has been detected in porcine cumulus cells using an *in situ* hybridization technique [16], while the latter has never been shown to be expressed in porcine ovaries. We identified connexins 43, 45 and 60 as the main connexins present in the porcine cumulus oocyte complex. Cx43 is strongly expressed in cumulus cells, Cx60 in the oocyte, and Cx45 in both the oocyte and cumulus cells.

The identification of the connexins involved is important, since by interacting to form heterotypic and heteromeric channels, different connexins can produce wide variations in gap junction permeability [13]. Connexins 43 and 45 are known to be involved in heteromeric and heterotypic gap junction structure. When these two connexins are present in the same cell GJC and gap junction size are usually decreased [58], [59], [60], [61]. Since these connexins are the main connexins expressed in cumulus cells, we examined variations in their expression during the early stages of IVM. We sought to determine in particular whether or not expression is regulated by gonadotropins and consistent with previously described modulation of GJC during the first hours of IVM [45]. The results of our previous experiments showed that Cx43 up-regulation is regulated at the transcriptional stage during the first hours of IVM and that transcription is required for increased GJC during the first 18 h of IVM [54]. We note with interest that in the rat, luteinizing hormone is involved in the inhibition of Cx43 transcription [62], [63]. In a rat granulosa cell line, follicular stimulating hormone can up-regulate the transcription of Cx43 and induce an increase in electrical coupling between granulosa cells [64]. Transcription of Cx43 has been shown to correlate with GJC in numerous cell types including porcine COCs [54], [65], [66], [67]. In the present study, we showed that Cx43 transcription is up-regulated at 4.5 h of IVM in a gonadotropin-dependent manner. Indeed, eCG and hCG act synergistically to increase Cx43 expression during this time period, while eCG or hCG alone have no effect and Cx43 expression remains stable. The Cx43 transcription profile in presence of eCG and hCG is similar to the GJC profile observed using FRAP in our previous study [45], which shows an increase in GJC during the first 4.5 h of IVM and a drastic decrease thereafter. However, this similarity between transcription profile and GJC was not seen when eCG or hCG was used alone. Indeed, in these conditions, GJC drop after 4.5 h of IVM was observed in the presence of eCG, while in the presence of hCG there was no drop after 4.5 h [45]. These results suggest that Cx43 transcription is active but do not seem to be the limiting factor regulating gap junction permeability between cumulus cells during the initial hours of IVM. Our previous results suggested that de novo transcription of Cx43 is needed for an initial increase in GJC [54], while our current results show that up-regulation or down-regulation of connexin 43 transcription is not involved in the subsequent regulation of GJC between cumulus cells.

Transcription of Cx45 remained at a low level during the first hours of IVM, independently of the presence of gonadotropins, making it unlikely that Cx45 transcription is involved in the modulation of GJC, which was previously found gonadotropin-dependent [45]. Moreover, the Cx45 transcription profile was not consistent with the GJC profile, making the involvement of Cx45 transcription in GJC regulation unlikely during the first hours of IVM.

Since Cx43 is the major connexin composing gap junctions in porcine cumulus cells, we examined its pattern of expression during the IVM period, in order to determine whether or not these dynamics could regulate gap junction number and hence GJC during meiotic resumption. Gonadotropin-mediated regulation of Cx43 protein expression during follicular growth and IVM has been demonstrated previously [19], [54], [62], [68], [69], [70], [71], [72]. Decreased Cx43 protein expression also appears to be involved in decreasing gap junction permeability between numerous cell types [65], [73], [74], [75], [76]. Since we have demonstrated that both eCG and hCG strongly increased Cx43 protein expression during the first few hours of IVM, while subsequent Cx43 protein down-regulation occurred only in the presence of eCG, thus corroborating our previous FRAP experiment [45], we explain the mechanism through which GJC is regulated during IVM in terms of increasing and decreasing the total number of Cx43 molecules in cells, thus varying the number of gap junctions and hence GJC flow rate. The monoclonal antibody predicted to work in pig and used to detect Cx45 unfortunately produced multiple bands in the Western blots (data not shown), making it impossible to estimate Cx45 protein expression and hence its possible involvement in GJC modulation.

Connexin localization could also play a role in regulating GJC. It has been shown that aberrant localization of gap junctions is involved in many diseases, in particular cancer and cardiac arrhythmia [3]. Indeed, aberrant localization of connexin 43 is involved in down-regulation of GJC and subsequent deregulation of cell growth [77], [78]. Many tumours are derived from a primary tumour in which the cells lost the ability to communicate, due to a lack of gap junction plaques, resulting in the proliferation of cancerous cells [79], [80], [81]. We examined Cx43 localization in sections of COC cultured in conventional maturation medium containing eCG and hCG, conditions under which Cx43 protein expression (based on Western blot) and GJC had the same pattern of variation, increasing for the first 4.5 h and then decreasing. This pattern was visible in the COC sections, although localization of the faint amounts of Cx43 at 0 h was not an easy task. However, the immunolabelling for Cx43 at 0 h does not form large aggregates and is not exclusively localised in the periphery of cumulus cells. The aggregates visible at the cumulus cell boundaries at 4.5 h of maturation are unequivocal and suggest the presence of functional gap junction plaques. This result is consistent with our previous report showing high-volume GJC at 4.5 h of IVM [45]. After 8.5 h of IVM, Cx43 is less visible in the COC sections and no longer clearly partial to cumulus cells, although it is more difficult to localize Cx43 in any case, since the cumulus cells have started to expand at this point. This result is nevertheless consistent with our previous FRAP data showing reduced GJC at 8.5 h of IVM [45]. This experiment provides evidence that localization of Cx43 contributes, along with Cx43 protein down or up regulation, to regulation of GJC.

Based on its elimination by the inhibitor MG132, the degradation of Cx43 suggested by densitometry at 8.5 h time point was attributed to the proteosomal complex, which along with ubiquination is an important mechanism for quickly modifying the amount of functional gap junctions in many cell types [82]. Connexin turnover is rapid, with half-lives ranging from 1.5 to 5 h [83], [84], [85], [86], [87]. Since the FRAP experiment with MG132 did not corroborated a role for the proteasome in GJC, we examined the localization of Cx43 in COCs incubated with the inhibitor. The differential result shows that GJC might be reduced due to a changed pattern of Cx43 localization (agglomerate in Figure 7), while the amount of Cx43 present remains high (Figure 6). However, it should be noted that MG132 is not a specific inhibitor of Cx43 degradation via the proteasomal complex, but rather a general inhibitor, and may have a broad impact on COC physiology by inhibiting the degradation of other proteins, which may explain the absence of cumulus expansion in its presence. It has been shown in many cell types that MG132 can act on numerous cell-signalling proteins and on hence signalling cascades that determine cell fates [88], [89], [90], [91], [92]. Proteasome inhibitors are currently used in anti-cancer chemotherapy as anti-metastatic agents (reviewed in [93], [94]).

In conclusion, we have described new mechanisms involved in the complex regulation of dynamic changes in connexin expression, degradation and localization in swine COC during the early stages of IVM. Dynamic modulation of Cx43 protein largely correlates with GJC during IVM. Understanding how GJCs are regulated during the first several hours of IVM is crucial for better understanding the mechanisms that affect oocyte maturation and thus oocyte competence. The mechanism of this dynamic regulation, especially during IVM, is still not fully understood. The three-dimensional gap-FRAP technique that we have developed has contributed much to our efforts to decipher the regulation of GJC during IVM and thus oocyte maturation and the competence to develop into an embryo. This study provides evidence that gonadotropins clearly affect connexin 43 protein expression dynamics and that proteasomal degradation of connexin 43 contributes largely to the regulation of Cx43 protein available. As MG132 affect several aspect of COC physiology, we have to stay cautious of its contribution to the regulation of GJC between cumulus cells during the very first hours of IVM. However, there is line of evidence that MG132 may likely contribute to it. Connexin dynamics and GJC may in turn determine the development of an oocyte fully capable of giving rise to a healthy individual. Knowing the molecular modes of regulation of GJC and subsequently being able to use and/or modify them should provide a powerful tool in the quest to improve oocyte competence.
